# Supplementary material for: Predicting visual function by interpreting a neuronal wiring diagram
Source: Nature. 2024 Oct 2;634(8032):113–23. doi: 10.1038/s41586-024-07953-5 (PMC11446822; doi:10.1038/s41586-024-07953-5)
Supplement: Supplementary file 6 — CRF and ERF predictions for individual TmY4 and TmY9 cells. Analogous to Supplementary Data 3, but for TmY target types. Shown are the top four monosynaptic pathways, the strongest pathway passing through each of the top ten intermediary types (ranking from Extended Data Fig. 7), and the trisynaptic pathway Tm1–TmY–Dm3–TmY (see the section entitled Prediction of spatial normalization). [file 41586_2024_7953_MOESM6_ESM.zip › DataS4/TmY9qΓèÑ/720575940633612845.pdf]

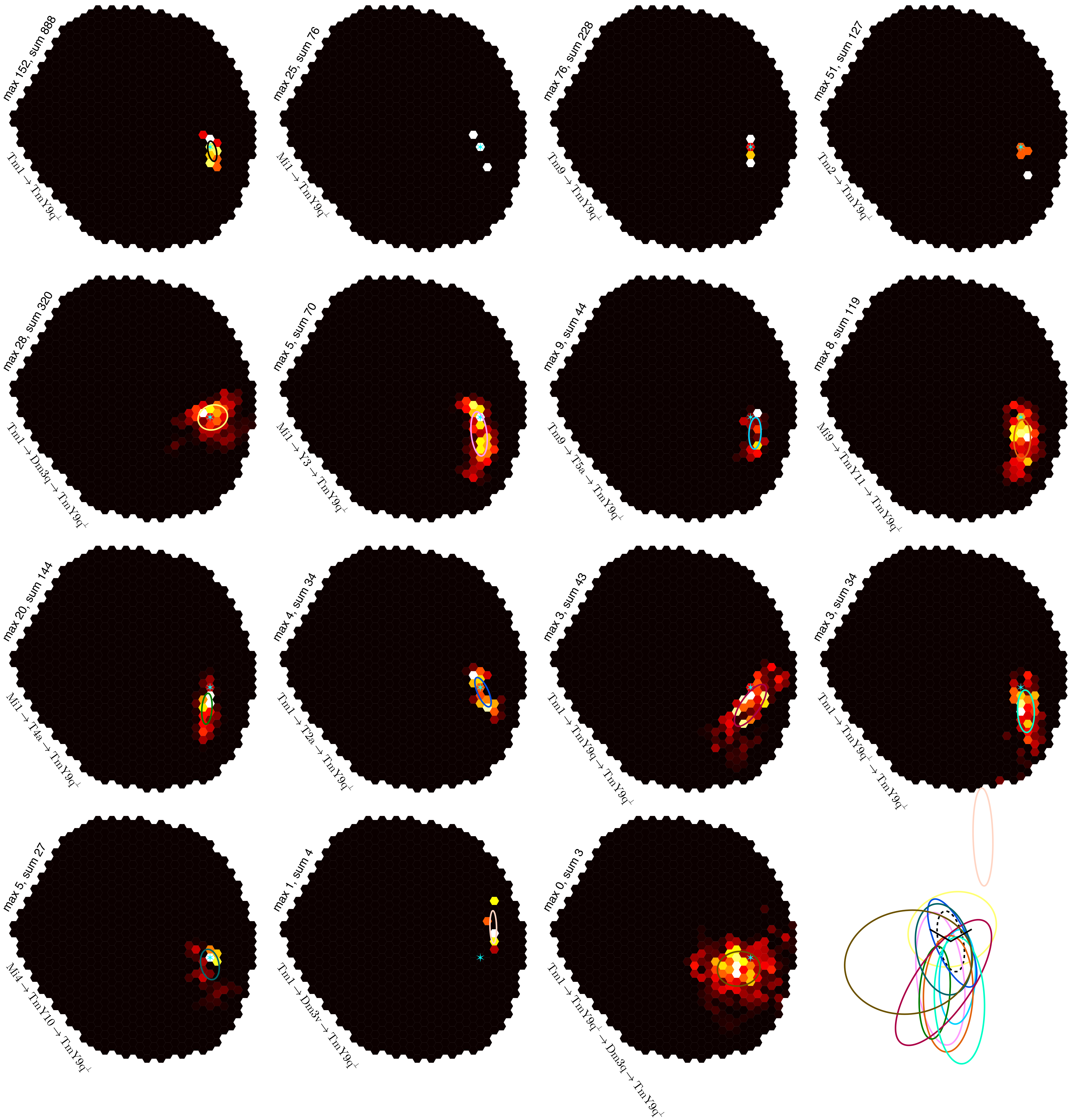

max 152, sum 888
Tm1 → TmY9q⊥
max 25, sum 76
Mi1 → TmY9q⊥
max 76, sum 228
Tm9 → TmY9q⊥
max 51, sum 127
Tm2 → TmY9q⊥
max 28, sum 320
Tm1 → Dm3q → TmY9q⊥
max 5, sum 70
Mi1 → Y3 → TmY9q⊥
max 9, sum 44
Tm9 → T5a → TmY9q⊥
max 8, sum 119
Mi9 → TmY11 → TmY9q⊥
max 20, sum 144
Mi1 → T4a → TmY9q⊥
max 4, sum 34
Tm1 → T2a → TmY9q⊥
max 3, sum 43
Tm1 → TmY9q → TmY9q⊥
max 3, sum 34
Tm1 → TmY9q⊥ → TmY9q⊥
max 5, sum 27
Mi4 → TmY10 → TmY9q⊥
max 1, sum 4
Tm1 → Dm3v → TmY9q⊥
max 0, sum 3
Tm1 → TmY9q⊥ → Dm3q → TmY9q⊥
